# Aspirin and clopidogrel resistance; a neglected gap in stroke and cardiovascular practice in Iran: a systematic review and meta-analysis

**DOI:** 10.1186/s12959-023-00522-2

**Published:** 2023-07-27

**Authors:** Mohammad Parsa-kondelaji, Hassan Mansouritorghabeh

**Affiliations:** 1 Experimental Hematology and Blood Banking, Mashhad University of Medical Sciences, Mashhad, Iran; 2 Central Diagnostic Laboratories, Mashhad University of Medical Sciences, Ghaem Hospital, Mashhad, Iran

**Keywords:** Aspirin, Clopidogrel, Drug resistance, Systematic review, Meta-analysis

## Abstract

**Objective:**

Antiplatelet drugs, such as Aspirin and Clopidogrel (Plavix) are effective in the primary prevention of thromboembolic events. They are commonly used to reduce the risk of recurrence of thromboembolism. The body’s hemostatic system responds differently to these drugs in different people. Resistance testing for aspirin and Clopidogrel is now recommended before starting antiplatelet therapy.

**Methods:**

A systematic literature search was performed on May 12, 2021, using the medical search engines PubMed, Scopus, and Web of Science, and the local databases SID and Magiran. After data extraction, a meta-analysis was performed using Comprehensive Meta-Analysis (CMA2) software. The I2 statistic was used to measure heterogeneity between estimates.

**Results:**

Among the 949 papers, Clopidogrel resistance was assessed in 136 patients and Aspirin resistance in 400 patients. The prevalence of Aspirin resistance was found to be 52.1% and the prevalence of Clopidogrel resistance was found to be 20.5%.

**Conclusion:**

It seems that in Iran, the issue of Aspirin and Clopidogrel resistance is suboptimally addressed. This pattern could also occur in other developing countries in the Middle East region.

## Introduction

**Fig. 1:**
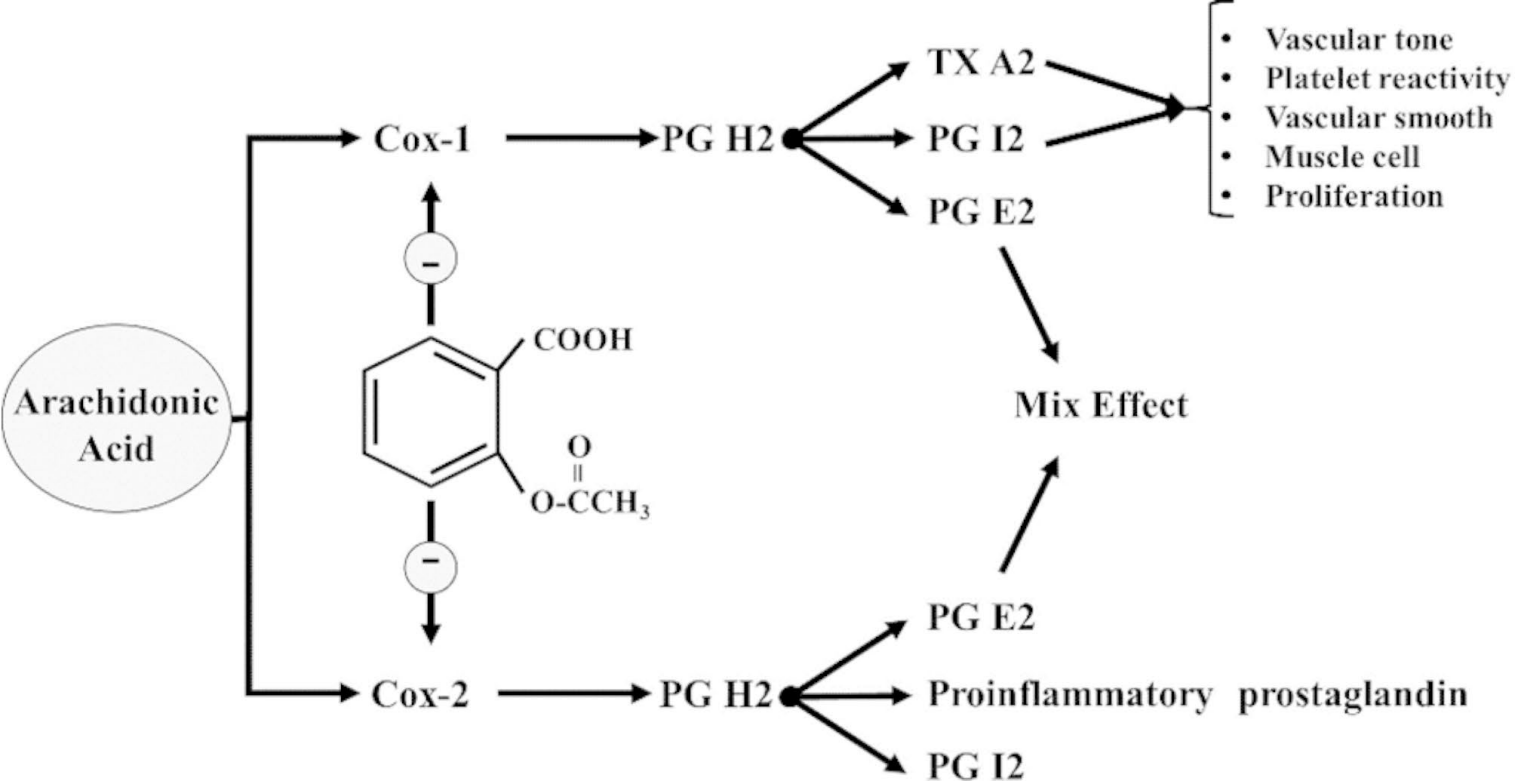
The mechanism of Aspirin actions through inhibition of cyclooxygenase-1 & 2 Cox: cyclooxygenase, PG: prostacyclin, TXA: Thromboxane A2

According to the World Health Organization (WHO), cardiovascular disease (CVD) accounts for approximately 31% of all deaths worldwide. In 2015, nearly 17 700 000 people died as a result of CVD. Most deaths from CVD deaths occurred in middle and low-income countries worldwide. There is no detailed article on CVD deaths in the Eastern Mediterranean region, which is home to approximately 580 million people [1, 2]. Aspirin was initially prescribed as an analgesic and antipyretic drug. Today, Aspirin is one of the most popular anti-platelet drugs for the prevention of thrombosis prevention [3]. More than 50 million people around the world take it regularly. The annual intake of aspirin is estimated to be about 40 000 tons [4]. Aspirin has been included in the list of important drugs for major health care systems at WHO due to its therapeutic efficacy, cost-effectiveness, and safety requirements [5]. The primary pharmacological action of aspirin is the suppression of the synthesis of thromboxane and prostaglandins. Synthesis of thromboxane and prostaglandin requires cyclooxygenase (COX) (Fig. 1). It is worth noting that aspirin targets cyclooxygenase COX-1 more strongly than COX-2. In other words, higher doses of Aspirin are required to inhibit COX-2, since Aspirin has a 170-fold lower potency for COX-2 [6]. Taking 100 mg of aspirin completely produces this effect. As a result, Aspirin inhibits platelet activation by inhibiting COX-1 at (529 Ser), and COX-2 at (516 Ser) sites [7]. Since platelets have a half-life of about 7 days and Aspirin has a half-life of 15–20 min, Aspirin may suppress platelets throughout the lifespan of the affected platelets [8–10].

**Fig. 2:**
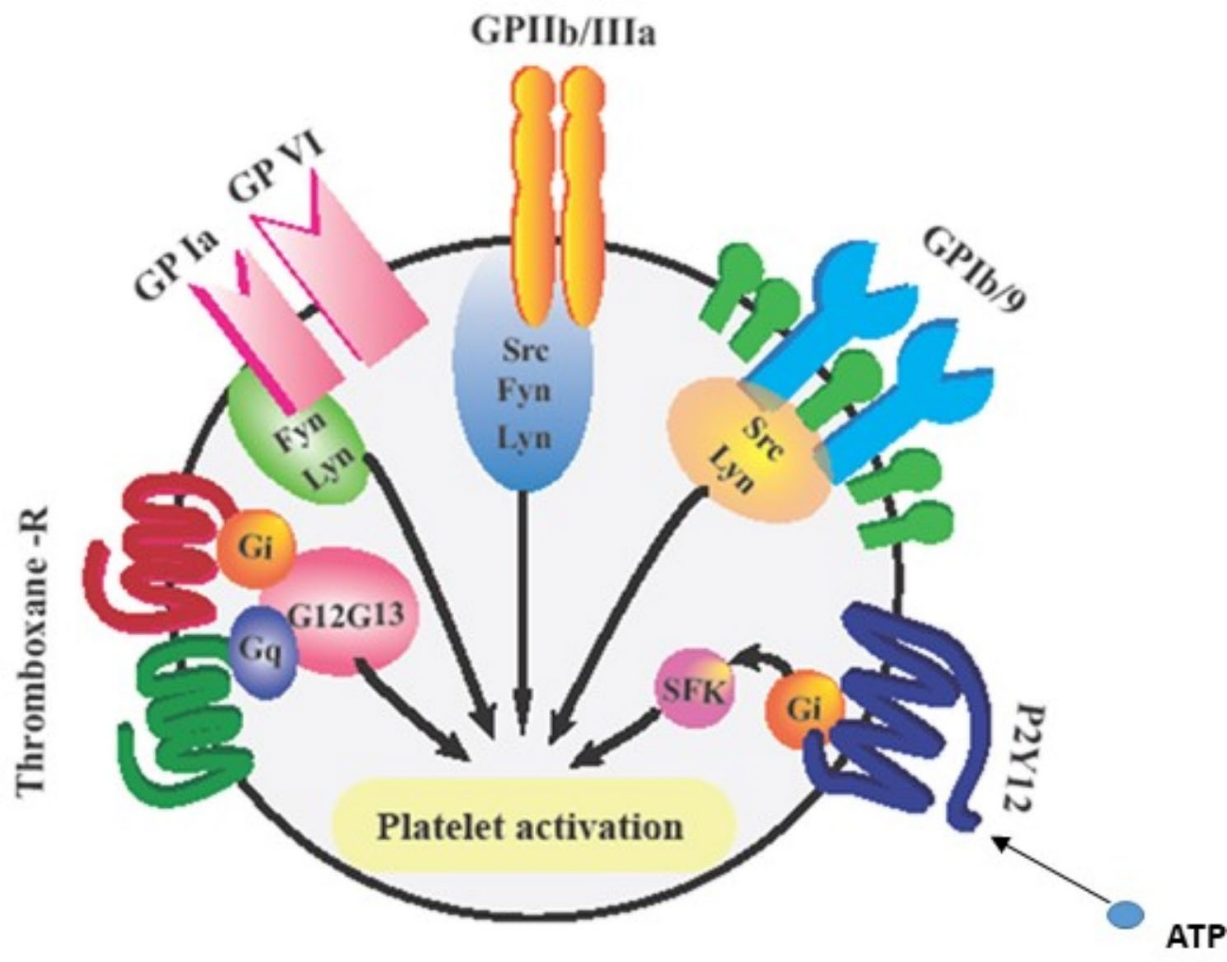
The schematic picture of various surface glycoproteins on platelets and their activation pathways, which lead to activation of platelets

Clopidogrel bisulfate, marketed as Plavix, is a second-generation, thienopyridine drug first marketed in 1991 [11]. It is used alone or in combination with Aspirin to reduce the risk of atherosclerotic events, such as myocardial infarction and stroke [12]. Clopidogrel acts as a P2Y12 antagonist. P2Y12 chemoreceptors are mainly located on the surface of platelets. When platelets are stimulated, ADP is released from their dense granules, which enhances the platelet response to weak agonists. The platelet receives an activating signal when ADP binds to P2Y12 (Fig. 2). P2Y12 receptors on platelets are inhibited by Clopidogrel, and the platelet response to ADP is also inhibited [13, 14].

Aspirin resistance has been defined as laboratory or biochemical resistance, i.e., the development of a laboratory platelet response despite the use of Aspirin that does not block platelet activity [15]. Resistance to Plavix as a drug that does not fulfill its intended purpose of inhibiting platelet function is considered Plavix resistance. Indeed, a weak response to clopidogrel, cause the detection of a change in ADP induced platelet activity compared to the reference point [16]. In the last decade, medical researchers have focused their research on aspirin resistance (AR) and clopidogrel resistance (CR) (Table 1). The prevalence of AR in patients treated with aspirin in different countries ranges from 5 to 60%, according to the available literature [17]. The prevalence of CR ranges from 17 to 25% in different populations, with an average of 21% [18]. It appears that emphasizing the importance of AR and CR paves the way for improved prevention of thrombotic events in the general population. It is important to determine an appropriate individual Aspirin dose to ensure optimal drug efficacy over time [19]. It has been reported that CR has not been extensively investigated in Asian communities to date [20]. This systematic review and meta-analysis was conducted on published papers on AR and CR in Iran as an example of a developing country in the Middle East region to portray understanding of the importance of this issue in clinical practice in developing countries.

**Table 1:** The characteristics of the included studies in the meta-analysis

The first author	Investigated drug	Publication Date	Type of study	Study location	Types of diseases	Test	Number of the patients	Age (year)	Sex Female (%)	Prevalence of resistance (%)	Hypertension (%)	Hyperlipidemia (%)	Diabetes (%)	Ref.
Sadeghi, M	Aspirin	2012	Retrospective study	Isfahan (center)	ischemic heart disease	Thromboxane B2 level in urine (ELISA)	170	60.42 ± 8.43	79 (46.5)	75.3%	58.8%	47.1%	25.3%	25
Kojuri, J	Aspirin	2010	Crossover	Shiraz (center)	percutaneous coronary angioplasty	light transmission aggregometry	106	56.07 ± 10.13	66 (62.26)	30.2%	54.7%	84.9%	12.2%	24
Eskandarian, R	Aspirin	2012	Cross-sectional	Semnan (north eastern)	coronary artery diseases	urinary 11-dehydro-thromboxane B.	124	61.8 ± 9.7	50 (40.3)	49.2%	33.8%	NR	28.2%	23
Aghajania, HM	clopidogrel	2018	Cross-sectional	Tehran (center)	angioplasty	light transmission aggregometry (LTA)	105	60.30 ± 12.2	64 (61%)	24.76%	65.71%	NR	20.95%	21
Aghajani, HM	clopidogrel	2013	Before-and-after	Tehran (center)	undergoing angioplasty	light transmission aggregometry (LTA)	31	59 ± 13	12 (38.7)	13%	54.8%	NR	19.4%	22

Ref: reference, NR: not reported

## Materials and methods

### Strategy of search and criteria

A systematic search was performed in PubMed, Scopus, and Web of Science databases to find articles on the prevalence of Clopidogrel and Aspirin resistance. Moreover, local databases such as SID and Magiran were searched to detect local articles. The used keywords were as follows: “Aspirin” OR “Acetylsalicylic acid” OR “Aspirin resistance” OR “Acetylsalicylic acid resistance” OR “platelet resistance” OR “Clopidogrel” OR “Clopidogrel resistances” OR “Plavix” OR “Plavix resistances” OR “antiplatelet” OR “urinary thromboxane disease” OR " platelet function assay 100” OR " platelet function assay 200” OR " Rapid platelet function assay " OR " flow cytometry " OR " light transmission aggregometry " OR " multiple electrode aggregometry " OR “96-well plate aggregometry " OR " cone and platelet analyzer (Impact-R)” OR " Plateletworks " OR " single platelet counting system " OR " vasodilator-stimulated phosphoprotein phosphorylation " OR " VASP “AND “Iran”. The equivalent words in Persian were searched in the cited local search engines. The literature search ended on May 12, 2021. After the literature search, all retrieved articles were entered into EndNote X7 reference manager software to perform the review procedures. In the first stage, duplicate articles were deleted. Then, the titles and abstracts of all papers were independently reviewed by the two authors.

### Inclusion and exclusion criteria

Studies were included if they met the following criteria in this systematic review: (1) published in English or Persian languages, (2) assessed the prevalence of AR or CR, (3) The study was performed on the Iranian’s population, (4) Relevant paper was among cross-sectional, case-control, before and after, and cohort or interventional studies.

The excluding criteria were: (1) Studies with insufficient information, (2) The review articles, meta-analysis, case reports and letter to the editor were excluded from the analysis.

### Quality assessment of selected papers

Two reviewers (M. P. and H. M.) independently assessed the quality of the studies. The each selected study was scored from 0 to 10. By summing the scores the risk of bias was determined. The final score of 0–3 was low, 4–6 was moderate, and 6–10 was high risk. Any conflict was clear in a joint meeting. The Joanna Briggs Institute (JBI) Appraisal Tool (check list) was used to assess the quality of included studies.

### Data extraction and statistical analysis

We extracted information from the articles into the Excel database. Data extraction included; the first author’s name, year of publication, study location, type of study, number of patients, patient age and sex, and the prevalence of AR or CR.

Comprehensive Meta-Analysis (CMA2) software was used to perform this meta-analysis. The prevalence rates of the studies were combined using a weighted average. The I2 statistic was used to measure heterogeneity between estimates. An increase in the degree of heterogeneity was described as I2 > 50%. In the meta-analysis, the random effect model was used to perform the analysis. The funnel plot test was used to assess publications bias.

## Results

### Selection and characteristics of the articles

**Fig. 3:**
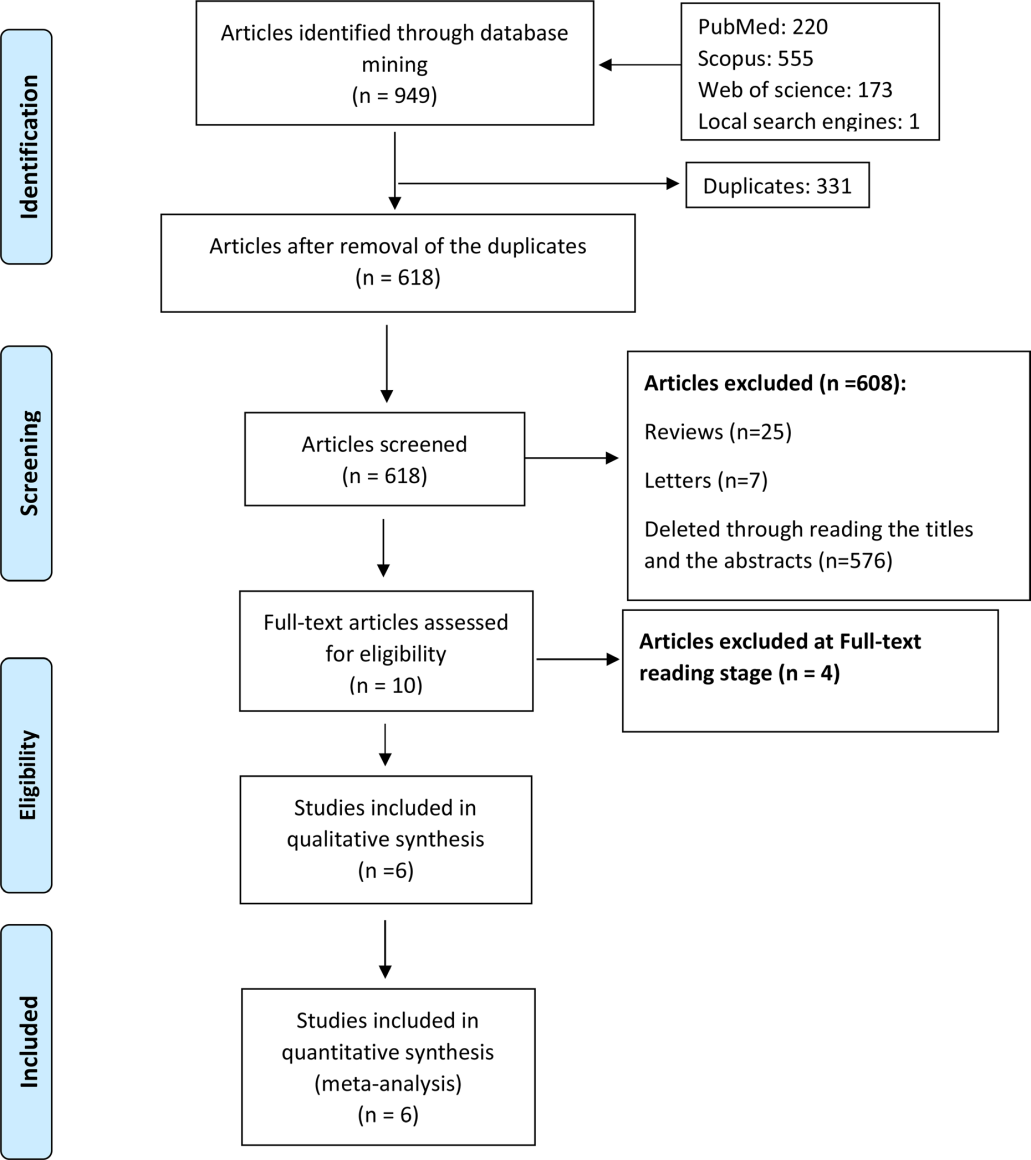
Flow-chart that is describing the selection of literatures through the strategy of including and excluding criteria

After reviewing the literature according to the search strategy, 949 articles were found, including 220 articles from PubMed, 555 articles from Scopus, 173 articles from the Web of Science and one article from the local database SID and Magiran. After removing duplicates, there were 618 papers remained. The papers were then evaluated based on their titles and abstracts. Then, 25 review articles, seven letters to the editor, and 576 unrelated articles were removed. Finally, the full text of ten articles were reviewed, of which six were included in the systematic review (Fig. 3). Table 1 shows the characteristics of the included studies.

### Studies on CR

According to our literature review, there were two papers on CR, which investigated the prevalence of CR in 136 individuals [21, 22]. Therefore, a meta-analysis on this topic was not performed because there was only one paper. This study showed a mean of 20.5% for CR in the studied population.

### Studies on AR

**Fig. 4:**
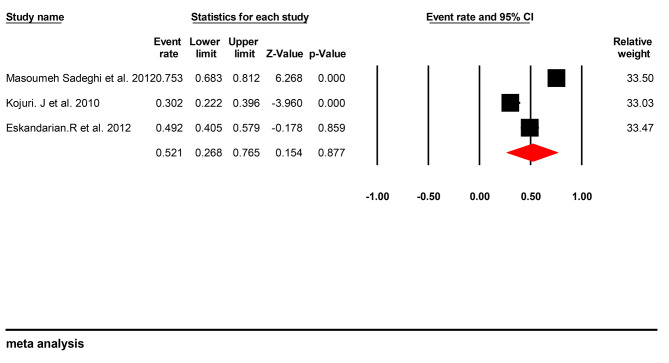
The forest plot of the Aspirin resistance prevalence in cardiovascular patients with its 95% confidence interval based on the random-effect model

**Fig. 5:**
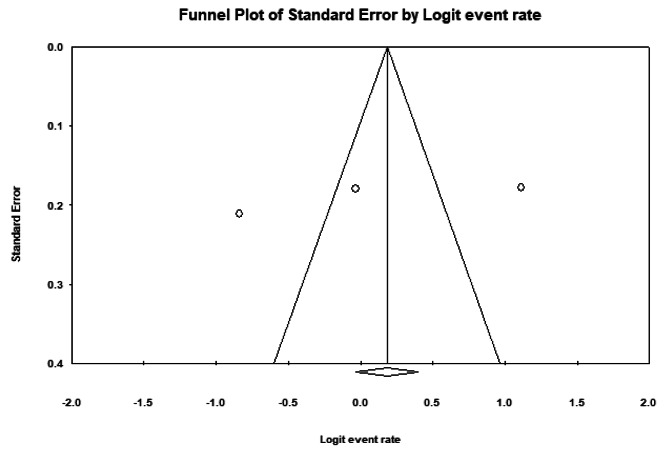
The Funnel plot of the studies included in the meta-analysis of Aspirin resistance

Three papers had investigated AR. A total of, 400 cardiovascular patients were studied in these studies [23–25]. There is a discrepancy between the number of retrieved papers on AR (four articles) and the number of papers included in the analysis (three articles). The overall prevalence of AR in patients with cardiovascular disease in the Iranian population was 52.1% ([95% CI 26.8–76.5], I2 = 96.16%, p: 0.877) (Fig. 4). A funnel plot was used to determine publication bias between studies (Fig. 5). There was no evidence of publications bias.

## Discussion

According to the present results, only three and two papers were published on AR and CR in Iran, respectively. In fact, there were four papers on AR, but one study was published twice [26]. Therefore, one of them was deleted from the analysis to avoid bias. According to the results of the current systematic review and meta-analysis, the prevalence of Aspirin and Plavix resistance in Iranian patients was 52.1% and 24.76%, respectively. Although, these results are consistent with the worldwide prevalence [25], the discrepancy between AR and CR may be attributed to the lower number CR publications compared with the higher number of AR publications. The lower number of publications on CR may be a source of bias in determining the true estimate of the CR community. Iran is a country with a population of about 84 000 000 people. It is the second most populous country in the Middle East. These results highlight the lack of attention given to this therapeutic area. In Turkey, a neighboring country, 78 articles have been published on AR as of 2017, which is second only to the United States in this field [25].

The prevalence of these drugs resistances is influenced by a variety of factors, such as clinical factors, cell biology factors, genetic factors (differences between countries and continents), age [27], gender (women benefit less from Aspirin use), and dosage [28]. Comparisons of AR and CR between different populations are difficult because of these criteria.

Despite the fact that this was a simple study, we could not obtain reliable results on the prevalence of AR and CR in the country due to the lack of adequate publications on AR and CR. Only four of 31 provinces in the country have conducted studies in this field. Furthermore, these publications cannot be representative of the entire population of these provinces because of limited number of patients studied and the large population of the country. Therefore, transferring these limited data to the entire country could lead to bias. Another limitation of this study was the different methods for detecting used to detect Aspirin resistance that were used. To detect Aspirin resistance, Ziaie et al. used urinary thromboxane B 2 (TXB 2) level [26], Kojuri et al. used the light transmission aggregometry method [24]., and Eskandarian et al. used the ELISA kit to detect urinary thromboxane B 2 (TXB 2) [23]. It goes without saying that a similar detection approach is preferred for mining a robust prevalence.

## Conclusion

According to the International Monetary Fund, Iran is a semi-developed country, with a GDP ranking of 38 out of 144 countries. Among the countries in the Middle East and North Africa, Iran ranks first in several health indicators, including low maternal mortality rate, low neonatal, infant, and under-5 mortality rate, and high life expectancy [29, 30]. Therefore, we chose Iran as a semi-optimistic health benefit as a template for determining the status of attention to AR and CR in the Middle East. It is likely that in other developing countries in the Middle East AR and CR receive similarly little attention. Despite their importance in cardiovascular practice, AR and CR received little attention in Iran, according to this meta-analysis. As a result, clinicians and researchers in these countries should pay close attention to this crucial point in the cardiovascular field. It should be recalled that cardiovascular diseases are the leading cause of mortality and morbidity worldwide, which underscores, emphasizing the importance of this recommendation. However, despite the discovery of AR and CR in earlier decades, it seems that these issues are still poorly addressed in this country, and further research is needed.

## Declarations

### Availability of supporting data

The data are available upon request by email from the corresponding author.

## Abbreviations

CMA: Comprehensive meta-analysis
WHO: World Health Organization
CVD: Cardiovascular disease
COX: Cyclooxygenase
AR: Aspirin resistance
Ser: Serotonin
CR: Clopidogrel resistance
TXB 2: Urinary thromboxane B 2

## References

[1] Gersh BJ, Sliwa K, Mayosi BM, Yusuf S (2010). Novel therapeutic concepts the epidemic of cardiovascular disease in the developing world: global implications. Eur Heart J.

[2] McAloon CJ, Boylan LM, Hamborg T (2016). The changing face of cardiovascular disease 2000–2012: an analysis of the world health organisation global health estimates data. Int J Cardiol.

[3] Awtry EH, Loscalzo J (2000). "Aspirin " Circulation.

[4] https://apps.who.int/iris/bitstream/handle/10665/325771/WHO-MVP-EMP-IAU-2019.06-eng.pdf Accessed 15 May 2021.

[5] Navaratnam K, Alfirevic A, Alfirevic Z (2016). Low dose aspirin and pregnancy: how important is aspirin resistance?. BJOG: An International Journal of Obstetrics & Gynaecology.

[6] Altaweel YAbd-Al, Hameed (2017). Aspirin resistance in acute ischemic non-cardioembolic stroke: frequency and clinical study. Int J.

[7] Crofford LJ (1997). COX-1 and COX-2 tissue expression: implications and predictions. J Rheumatol Suppl.

[8] Rowland M (1972). Absorption kinetics of aspirin in man follow oral administration of an aqueous solution. J Pharm Sci.

[9] Cattaneo M (2004). Aspirin and clopidogrel: efficacy, safety, and the issue of drug resistance. Arterioscler Thromb Vasc Biol.

[10] Du G, Lin Q, Wang J (2016). A brief review on the mechanisms of aspirin resistance. Int J Cardiol.

[11] Patti G, Micieli G, Cimminiello C, Bolognese L. The role of clopidogrel in 2020: A reappraisal. *Cardiovascular therapeutics*, 2020 (2020). 10.1155/2020/8703627 PMC7140149 32284734

[12] Mullangi R, Srinivas NR (2009). Clopidogrel: review of bioanalytical methods, pharmacokinetics/pharmacodynamics, and update on recent trends in drug–drug interaction studies. Biomed Chromatogr.

[13] Feher G, Feher A, Pusch G (2010). Clinical importance of aspirin and clopidogrel resistance. World J Cardiol.

[14] Wang TH, Bhatt DL, Topol EJ (2006). Aspirin and clopidogrel resistance: an emerging clinical entity. Eur Heart J.

[15] Perrier-Cornet A, Ianotto J-C, Mingant F, Perrot M, Lippert E, Galinat H (2018). Decreased turnover aspirin resistance by bidaily aspirin intake and efficient cytoreduction in myeloproliferative neoplasms. Platelets.

[16] Alakbarzade V, Huang X, Ster IC, McEntagart M, Pereira AC (2020). High on-clopidogrel platelet reactivity in ischaemic stroke or transient ischaemic attack: systematic review and meta-analysis. J Stroke Cerebrovasc Dis.

[17] Helgason CM (1994). Development of aspirin resistance in persons with previous ischemic stroke. Stroke.

[18] Bishopric NH. “Toward a genomic definition of aspirin resistance.“ (2013): 1277–9. 10.1016/j.jacc.2013.06.024 23850916

[19] Qureshi Z, Alex R, Hobson (2013). Clopidogrel “resistance”: where are we now?. “ Cardiovasc Ther.

[20] Hidayat R, Nabilah RA, Rasyid A (2022). Clopidogrel resistance among ischemic stroke patients and its risk factors in Indonesia. Acta Med academica.

[21] Aghajani MH, Kobarfard F, pouzhia Shojaei S (2018). The impact of Clopidogrel Resistance on Clinical Outcome of iranian patients undergoing percutaneous coronary intervention. Iran J Pharm research: IJPR.

[22] Aghajani M, Haji et al. “Resistance to Clopidrogrel among Iranian patients undergoing angioplasty intervention.“ Iran J Pharm research: IJPR 12 (169), (2013). PMC3813374 24250685

[23] Eskandarian R, Darabian M, Heshmatnia J, Ghorbani R. Acetyl salicylic acid resistance in patients with chronic stable angina and the correlation with coronary risk factors. Saudi Med J, 39–43 (2012). 22273646

[24] Kojuri J, Mahmoody Y, Sabegh BZ, Jannati M, Mahboodi A, Khalili A (2010). Dose-related effect of aspirin on laboratory‐defined platelet aggregation and clinical outcome after coronary stenting. Cardiovasc Ther.

[25] Sadeghi M, Emami A, Ziyaei N, Yaran M, Golabchi A, Sadeghi A. Aspirin resistance and ischemic heart disease on iranian experience. Adv biomedical Res 1(33), (2012). 10.4103/2277-9175.99345 PMC3507033 23210092

[26] Ziaie N, Sadeghi M, Akhlaghi A, Pirhaji O, Yaran M, Pourmoghadas M. Aspirin resistance status as determined by urinary thromboxane B 2 (TXB 2) level in patients with ischemic heart disease and its relationship with severity of coronary artery disease. J Isfahan Med School 28(116) (2011).

[27] Al-Jabi SW (2017). Global trends in aspirin resistance‐related research from 1990 to 2015: a bibliometric analysis. Basic Clin Pharmacol Toxicol.

[28] FitzGerald R, Pirmohamed M (2011). Aspirin resistance: effect of clinical, biochemical and genetic factors. Pharmacol Ther.

[29] Guirgis M, Thompson P, Jansen S (2017). Review of aspirin and clopidogrel resistance in peripheral arterial disease. J Vasc Surg.

[30] Mirzaei H, Abdi Z, Ahmadnezhad E (2020). Health Status in the Islamic Republic of Iran, Middle East and North Africa Countries: implications for Global Health. Iran J Public Health.

